# Evidence-informed policy formulation and implementation: a comparative case study of two national policies for improving health and social care in Sweden

**DOI:** 10.1186/s13012-015-0359-1

**Published:** 2015-12-08

**Authors:** H. Strehlenert, L. Richter-Sundberg, M. E. Nyström, H. Hasson

**Affiliations:** Department of Learning, Informatics, Management and Ethics, Medical Management Centre, Karolinska Institutet, SE 171 77 Stockholm, Sweden; Department of Public Health and Clinical Medicine, Epidemiology and Global Health, Umeå University, SE 901 87 Umeå, Sweden; Center for Epidemiology and Community Medicine, Stockholm County Council, SE 171 29 Stockholm, Sweden

**Keywords:** Policymaking, Policy analysis, Soft laws, Governance, Health policy, Implementation, Stakeholders, Advocacy Coalition Framework

## Abstract

**Background:**

Evidence has come to play a central role in health policymaking. However, policymakers tend to use other types of information besides research evidence. Most prior studies on evidence-informed policy have focused on the policy formulation phase without a systematic analysis of its implementation. It has been suggested that in order to fully understand the policy process, the analysis should include both policy formulation and implementation. The purpose of the study was to explore and compare two policies aiming to improve health and social care in Sweden and to empirically test a new conceptual model for evidence-informed policy formulation and implementation.

**Methods:**

Two concurrent national policies were studied during the entire policy process using a longitudinal, comparative case study approach. Data was collected through interviews, observations, and documents. A Conceptual Model for Evidence-Informed Policy Formulation and Implementation was developed based on prior frameworks for evidence-informed policymaking and policy dissemination and implementation. The conceptual model was used to organize and analyze the data.

**Results:**

The policies differed regarding the use of evidence in the policy formulation and the extent to which the policy formulation and implementation phases overlapped. Similarities between the cases were an emphasis on capacity assessment, modified activities based on the assessment, and a highly active implementation approach relying on networks of stakeholders. The Conceptual Model for Evidence-Informed Policy Formulation and Implementation was empirically useful to organize the data.

**Conclusions:**

The policy actors’ roles and functions were found to have a great influence on the choices of strategies and collaborators in all policy phases. The Conceptual Model for Evidence-Informed Policy Formulation and Implementation was found to be useful. However, it provided insufficient guidance for analyzing actors involved in the policy process, capacity-building strategies, and overlapping policy phases. A revised version of the model that includes these aspects is suggested.

**Electronic supplementary material:**

The online version of this article (doi:10.1186/s13012-015-0359-1) contains supplementary material, which is available to authorized users.

## Background

Evidence has come to play a central role not only in evidence-based medicine but also within health policy [1, 2]. Research on evidence-based policy has often started with the assumption that the use of more research would lead to a better policy [2, 3]. Recently, a call was made for studies aiming to understand the complex processes behind policy change [2]. Several authors have suggested that an unprejudiced and more explorative approach would be useful [2, 4–6]. This would imply more focus on understanding the processes behind using evidence and taking into consideration the contextual factors.

Policymakers tend to interpret evidence in a broad sense and to use other types and sources of information besides research evidence [7]. Non-research evidence has been defined as the views of local stakeholders, including expert and professional opinions, values and traditions, lobbyists and pressure groups, and the particular pragmatics and contingencies of the policy situation [8]. The term “evidence-informed policy” has been suggested to reflect this variety of sources [4]. Oxman et al. defined evidence-informed policymaking as an approach that aims to ensure that decision-making is informed by the best available research evidence in a systematic and transparent way [9].

It has been argued that in order to fully understand the policy process, the analysis should also include policy implementation [2]. Most prior studies within the area of evidence-based policy (with some exceptions, e.g., [10]), have not conducted systematic analysis of implementation. Several authors have suggested that using literature from both implementation science and policy implementation research could be beneficial for understanding policy implementation processes [11, 12]. Scholars within political science have focused on governance and inter-organizational relationships [13]. Governance deals with creating conditions for collective actions. This kind of governance (as opposed to legislative governance) is synonymous with the term “soft law” [14]. The Advocacy Coalition Framework (ACF) proposes that multiple actors who are motivated by their beliefs form advocacy coalitions and attempt to influence policy by using multiple resources, strategies, and institutional arenas [15]. Categories of resources that can be employed by coalitions include access to legal authority, public opinion, information, mobilizable troops, financial resources, and skillful leadership [16].

### Conceptual model for evidence-informed policy formulation and implementation

**Fig. 1:**
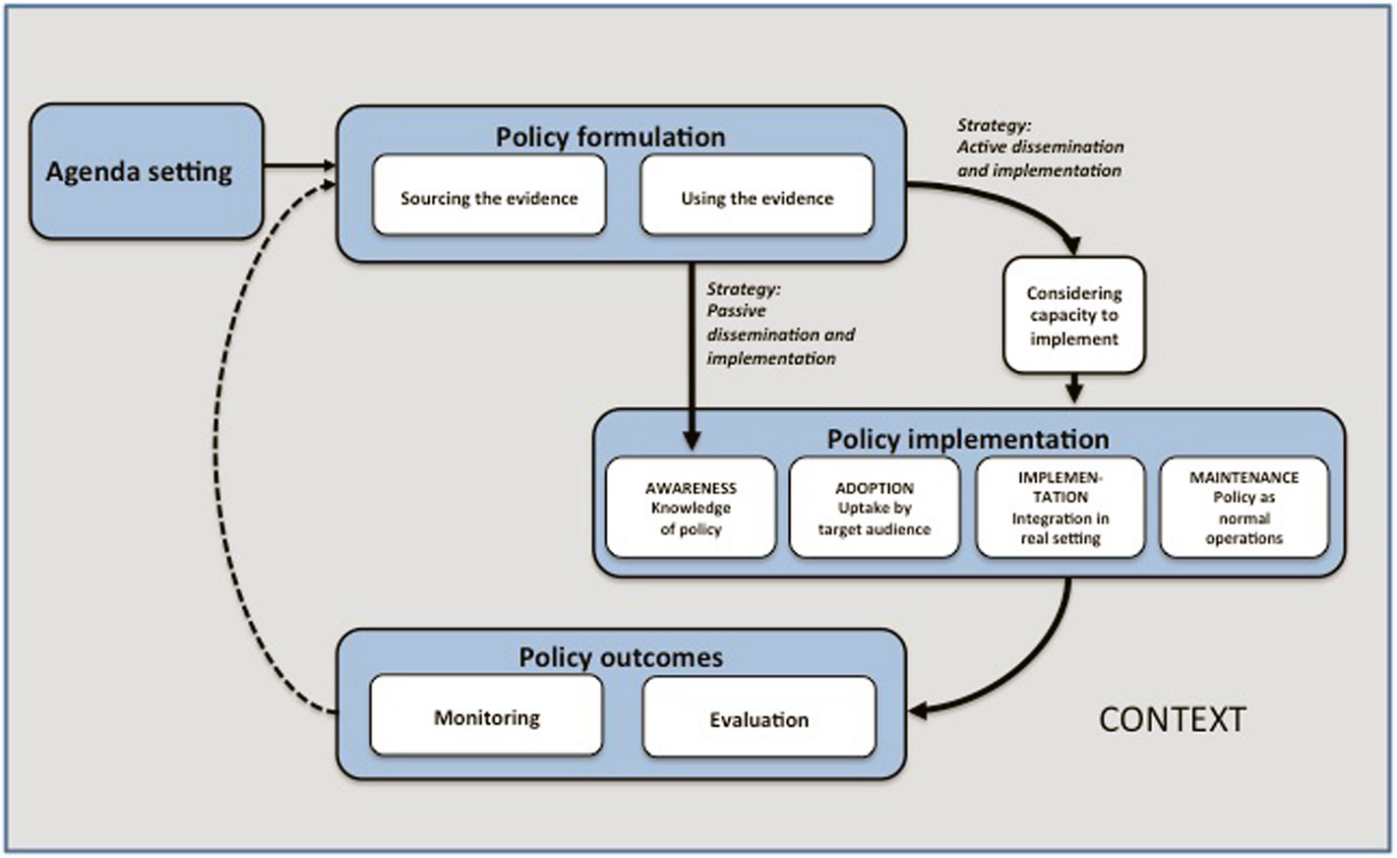
Conceptual Model for Evidence-Informed Policy Formulation and Implementation. Developed from the frameworks by Bowen & Zwi [1] and Dodson et al. [23]

In the current study, a conceptual model for analyzing evidence-informed policy formulation and implementation was developed. We combined central features of a framework for evidence-informed policymaking [4] and a framework for policy dissemination and implementation [17] in order to cover the whole policy process (Fig. 1). The model mirrors the classical illustration of the policy process consisting of the following phases: agenda setting, policy formulation, policy implementation, and evaluation [18].

Bowen and Zwi [4] proposed that the *use of evidence* involves active interpretation and balancing of scientific knowledge in relation to other types of knowledge. Target audiences’ *capacity to implement* deals with target individuals’, organizations’, and systems’ capacity to carry out the policy objectives. Dodson et al. [17] suggested that policymaking agencies need to make decisions on how to disseminate and implement the policy. Two basic alternatives exist: a passive strategy implying mere dissemination and a more active approach with outreaching strategies to influence awareness, adoption, implementation, and maintenance of the policy. *Adoption* includes the target audiences’ decision to implement the policy [17]. The *implementation* stage involves activities to improve knowledge and skills, and facilitation of the change process. *Maintenance* focuses on ensuring the continued use of the policy as part of organizational operations. The *policy outcomes* involve monitoring of whether the policy is being implemented as planned, as well as impact evaluation aiming to establish a causal relationship between the policy and changes in outcomes [19]. Bowen and Zwi [4] also suggested that the context influences the policymaking process and that evidence needs to be contextualized for effective policymaking. They define *context* as the setting in which the policy is developed and implemented, consisting of political, social, historical, and economic elements, as well as the healthcare system and service context.

The purpose of this study was to explore and compare two policies aiming to improve health and social care in Sweden and to empirically test the Conceptual Model for Evidence-Informed Policy Formulation and Implementation.

## Methods

### Study design

This study draws on two empirical projects. A longitudinal, comparative case study approach was applied. Case study 1 focused on the development and implementation of national guidelines for methods of preventing disease that took place between 2007 and 2014 [20]. Case study 2 explored the development and implementation of a national policy aiming to improve the quality and coordination of care for the most ill older people, between 2009 and 2014 [21] (for descriptions of the cases, see Additional file 1 and for description of the healthcare system and actors, see Additional file 2). These policies share some key characteristics: (1) focus on health and social care, (2) emphasis on prevention, (3) broad scope, and (4) multidisciplinary target audiences, including all regional and local authorities and multiple professional groups. The cases differed mainly regarding what actors were involved.

### Data collection

For both cases, data covering the whole policy period was collected (Case 1 2007–2014; Case 2 2009–2014). Public and non-public documents were collected for all policy phases (Case 1 *n* = 18; Case 2 *n* = 70). Semi-structured interviews were conducted with a purposive sample of 22 key stakeholders between 2009–2014 (Case 1 *n* = 10) and 2012–2014 (Case 2 *n* = 12). The interviews focused on how the policy was formulated, what strategies and activities were used, and the actors involved. The interviews (lasting between 45 and 90 min) were recorded and then transcribed. The informants were seen as proxies for their respective organizations at an institutional level. Forty-seven observations of meetings, seminars, and conferences were conducted between 2009–2014 (Case 1 *n* = 9) and 2012–2014 (Case 2 *n* = 38). The main purpose was to gain familiarity with the process and actors. In both cases, the observations were made by two researchers (Case 1 LRS and MN; Case 2 HS and MN). A structured protocol indicating time and the meeting’s pre-set agenda were used. Observational data contained aspects of the content (e.g., what was verbalized by whom in speech or writing) and the process (e.g., types of activities and procedures). Most observations were non-participatory, but a few observations also included feedback and discussions about preliminary results. The participatory observations enabled the researchers to ask follow-up questions based on previous observations and to validate results.

### Data analysis

The transcribed interviews were entered into software for qualitative analysis (Open Code 4.02, NVivo 10) and analyzed using a directed content analysis approach [22]. Data that could not be coded into the categories of the conceptual model formed new categories reflecting relevant aspects of the policy process. Information about key stakeholders, events and relevant process, and contextual factors were identified in the documents and observation protocols, compiled in chronological matrix for each case, and then coded using the stages in the conceptual model. The next step involved a synthesis to prepare a case record for each policy. Case description consistency was cross-checked between interviews and documents to ensure internal validity. Finally, key similarities and differences were discussed and identified. Ethical applications were sent to the Regional Ethics Committee in Umeå (Case 1, ref no. 2011-64-31M), which approved the study, and the Regional Ethics Committee in Stockholm (Case 2, ref no. 2011/5:11), which judged that the study had no ethical aspects to be considered.

## Results

### Case 1

#### Agenda setting

The initiative to develop the preventive services was raised as a political issue. There was a general positive development within public health, though there were some worrying trends concerning obesity and use of alcohol. National surveys indicated that there were large variations in preventive practices between the regions. In 2007, the National Board of Health and Welfare (NBHW) started to develop national guidelines with a disease-preventive scope that aimed to provide recommendations about treatments and methods, financial and organizational consequences of adhering to the guidelines, and indicators for assessment.

#### Policy formulation

##### Sourcing evidence

A structured process was used, regulated by NBHW’s steering documents, which standardize the process of searching, assessing, and prioritizing evidence. Expert groups consisting of researchers with expertise in each of the target areas conducted systematic literature searches. Relevant conditions (e.g., daily smoking) and interventions (e.g., advanced counseling) were identified and paired, and then the quality of evidence was assessed using the GRADE system.

##### Using evidence

High quality research evidence played a key part in the process. Based on the literature searches, the expert groups summarized effect sizes, methodological considerations, and generalizability of findings. This resulted in a selection of studies that were included in the remaining process. The prioritization group, consisting of 25 health professionals, was given the evidence, compiled and assessed by the expert groups. The prioritization group then formulated the policy recommendations based on group discussions and consensus decisions. Three criteria were used: the strength of the scientific evidence, cost-effectiveness, and ethical considerations.

#### Strategy for dissemination and implementation

The guidelines were launched in 2011. Though NBHW is not by default responsible for supporting guideline implementation, the government decided to employ an active strategy and gave NBHW an assignment and funding to support the implementation during 2011–2014. The main tasks were to organize the dissemination to the regional and local levels and to develop an interactive web-based platform on disease prevention.

#### Capacity to implement

During the last guideline development phase, NBHW arranged regional seminars to allow representatives of the target audiences to discuss guideline implications. Implementation challenges were identified, mainly because the guidelines were comprehensive and would affect all parts of the healthcare system. The policy implied a paradigm shift in emphasizing the preventive aspects of healthcare. The policy had organizational implications regarding steering documents, staff training, relocation, and coordination of resources and actors. A decision-maker at NBHW described this:

Most clinical guidelines focus only on a limited part of the healthcare services, but in this case we are talking about the entire healthcare system. […] We realized that this was going to be a challenge – to change the attitudes and ways of working among such a vast array of recipients and contexts. It was obvious that the need of support to succeed with the implementation was bigger than for other guidelines.

NBHW established regional networks, consisting of managers and healthcare developers, to share experiences and analyze implementation barriers and facilitators. The regular network meetings allowed NBHW to monitor the regional capacity and adapt the implementation support.

#### Implementation

##### Awareness

The process was described as a complex interplay of top-down and bottom-up processes between the government agency and the regional healthcare organizations. The guideline model implies an active strategy for raising awareness by involving stakeholders during the development of the guidelines. This was done through involvement of health professional organizations and via dialogue with healthcare decision-makers. By using the professional organizations’ existing infrastructures for communication, the implementation activities could start without delay. The aim was to gain support from healthcare professional actors and thereby increase the chances for positive reception among target audiences. A decision-maker at NBHW described this:

We are working through the health professional organizations. […] There were indications that we would have to change the attitudes among the target audiences, […] and in order to reach out it would be better if the health professional organizations acted as senders, rather than NBHW.

##### Adoption

NBHW offered funding to seven health professional organizations to launch implementation projects and to researchers, aiming to increase knowledge of how the guidelines could be adopted. The uptake of the policy was described as complex and slow, but as a potentially promising process. Barriers to adoption were inflexibility of the documentation systems and negative attitudes among parts of the health professional groups. No national performance-based grants were disbursed; instead, the regional authorities developed their own financial incentives.

##### Implementation

A core management group at the NBHW coordinated the implementation. They analyzed information to identify the support needed. Possible solutions were sought and developed together with stakeholders and actors in healthcare. NBHW also formed a reference group with representatives from national stakeholders (e.g., the Swedish Association of Local Authorities and Regions (SALAR), the Swedish Society of Medicine, and the National Food Agency). NBHW’s four key strategies were to (1) engage the health professional organizations, (2) support the managers and healthcare developers with knowledge to facilitate the change process, (3) create an arena for exchanging experiences (i.e., network meetings hosted by NBHW), and (4) support research on the implementation of disease prevention methods.

##### Maintenance

NBHW’s strategy was to place the responsibility for continued enforcement of the policy on the health professional organizations and the regional and local authorities. This was based on the idea that the professions and the healthcare decision-makers are best suited to judge the needs for and barriers to implementing the guidelines. NBHW is responsible for updating the guidelines every 3 to 5 years, repeating the development and dissemination procedure. Quality indicators for the guidelines were implemented in the patient record systems, and the aim was to connect these indicators to the annual national quality measurements and a national quality registry in the future.

#### Outcomes

The guidelines include a model to measure policy and implementation outcomes. The outcome measurement will show the proportion of patients who received a specific recommendation and the results. Nine indicators will be reported from patients’ medical records to NBHW. The results will be public and openly compared. However, the reporting of indicators encountered barriers in the medical record systems, and there were still ongoing development in June 2014.

### Case 2

#### Agenda setting

The need to improve care for older people was well acknowledged among all stakeholders, along with an awareness that evidence-based practices were not being applied systematically. Previous improvement initiatives had been difficult to evaluate, and there were large local variations in the quality and coordination of care for older people. Several related policies in health and social care including performance-based grants were being implemented by the government and SALAR. A policy resonating these interests was developed in 2010 in negotiations between the government and SALAR. The main goals, content, and actors were established at the start, but the annual renegotiations 2010–2014 enabled the parties to take the past year’s evaluation into account before making decisions for the next year. A member of the project management team at SALAR described this:

Initially, we [SALAR] did not like the conditional requirement about documentation of a management system for systematic quality work. […] But we yielded on that point, and the support to our members [to meet the requirement] has helped them. Now they are more aware of their decision-making structures, what regulations apply and what templates they should use… So I guess there have been some positive effects.

The ambition was to formulate a coherent policy comprising a few important improvement areas and to stick to these for the whole period while successively increasing the requirements for the performance-based grants.

#### Policy formulation

##### Sourcing evidence

This did not follow a structured or pre-defined process. In the first two agreements, both parties used their own experiences as well as their political and social values to negotiate the content. An expert at NBHW described this:

The choice of improvement areas eligible for performance-based grants was made through some kind of consensus procedure, by a small group of experts from the organizations involved. We tried to identify the main problems from a more value-based point of view and then we had to narrow down the list due to availability of data. There were important areas that needed improvement where we couldn’t find any suitable indicators or data.

SALAR, which was responsible for the administration of national quality registries, identified relevant registries (e.g., within palliative care) and suggested using them in the policy as a means for improving the older people care. National quality registries contain patient-level data regarding diagnoses, treatments, and outcomes. The use of the registries involved systematic assessments with validated instruments, evidence-based interventions, and follow-ups. In 2011, the government introduced a more structured strategy for sourcing information. A national coordinator for older people care was appointed to coordinate the government’s work, including the current policy. A problem investigation was conducted to serve as a basis for a more comprehensive agreement in the 2012 negotiations. Information was gathered through study visits, hearings, and interviews with stakeholders. A broad literature search was also conducted, mainly involving reports from national agencies.

##### Using evidence

Synthesized information from several sources was used. A pragmatic approach was used in the negotiations about the areas eligible for performance-based grants (preventive care, palliative care, dementia care, pharmacological treatment, and coordination of care). Knowledge and arguments were assessed regarding the perceived significance for improving care, scientific evidence, and the availability of suitable indicators.

#### Strategy for dissemination and implementation

Several actors were engaged, primarily SALAR, the national quality registries and the government, acting through the national coordinator at the Ministry of Health and Social Affairs (MHSA). SALAR was the main actor, responsible for engaging regional and local authorities, coordinating and supporting the implementation by organizing activities, and compiling and reporting on the results. The strategy also included capacity-building and establishing regional support structures (i.e., improvement coaches and higher-level managerial support).

#### Capacity to implement

Assessment of the capacity was based on the government’s and SALAR’s previous knowledge, and the hearings with experts and representatives of regions and local authorities and the professional organizations. The assessment resulted in specific capacity-building strategies, such as funding for support structures and conditional requirements for obtaining performance-based grants. The annual renegotiation of the agreement allowed for the successive development of the target audiences’ capacity to influence the content, performance levels, and implementation support.

#### Implementation

##### Awareness

SALAR had the formal responsibility to increase awareness, and strategic communication was given high priority. Initially, great emphasis was put on reaching out quickly with information. SALAR invited regional and local stakeholders to conferences and also used their established networks to disseminate the policy. The government and SALAR collaborated to raise awareness among target audiences and other groups, such as retiree organizations, for instance by conducting joint visits to all regions.

##### Adoption

The national performance-based grants provided a strong incentive for political and higher management levels to adopt the policy. SALAR’s direct contacts and ability to influence regional and local managers, as well as peer pressure among managers, were also important. A member of the project management team at SALAR described this:

No other organization in Sweden can reach out like SALAR, via our member networks. […] We can talk to [representatives from the regional and local authorities] behind closed doors and do whatever is needed to persuade them. No governmental authority can do that.

Benefits of evidence-based practices were used as arguments to spur on adoption among professionals. The core value of the policy, i.e., benefits for the individual patient, was emphasized. This was described as a strategic choice, since the need to improve older people care was the feature that all stakeholders strongly agreed upon.

##### Implementation

SALAR’s combined role as a partner in the agreement and as an interest organization for the target audiences enabled them to create pressure to implement the policy. Facilitative strategies were used extensively, with the focus on establishing regional support structures for the policy. Improvement coaches were hired in each region and SALAR supported the coaches via regular network meetings and a web-based interactive platform for sharing experiences and information. Collaborative teams of managers were formed in each region to drive the implementation from a managerial point of view. A program was initiated to inspire the teams to put action plans into practice. SALAR created arenas for education and sharing experiences for the management teams. SALAR also organized support to increase the regions’ capacity to analyze data. A web-based portal presenting indicators was developed, which made it possible to openly and continuously monitor the development. SALAR emphasized strategic communication in the implementation phase, stressing the value of every individual’s right to personalized, safe care. A large amount of information materials and a web-based tool for measuring older people’s experiences of care were developed. Numerous educational events were organized, and efforts were made to coordinate the activities with other ongoing policies to streamline the implementation. SALAR also participated in local and regional activities across the country. The quality registry actors played an important role. They provided support to the improvement coaches and users at the local level.

##### Maintenance

After the implementation, the disbursement of performance-based grants stopped, the project team at SALAR dissolved, and responsibility for continued implementation rested with the regional and local authorities. However, in the last agreement, the regional and local authorities were asked to present their plans for maintenance of the policy in order to qualify for the final year’s performance-based grants. SALAR arranged for the maintenance of the web-based tools, and the quality registries continue to support their users. Measurement of some of the indicators continues as parts of the annual national quality measurements.

#### Outcomes

Monitoring and feedback were central aspects of the policy. Indicators and target levels for performance-based grants were developed and refined over time for each improvement area. Initially, the monitoring focused on activities, but measures concerning outputs and outcomes were introduced successively. The web portal for outcome data enabled stakeholders to continuously view and compare results and to use the information for planning and systematic improvement work.

### Similarities and differences between the cases

In both cases, policymakers gathered information through their networks to assess the capacity to implement. Assessments showed that the target groups lacked the capacity to act on the policy without support and thus actions were taken to strengthen the capacity. The implementation strategies in the cases were also similar: dissemination through existing channels, interactive educational activities, and creating arenas for support and sharing experiences between regional and local implementers. As for policy outcomes, indicators were identified for monitoring, feedback, and comparison of results in both cases. The cases differed regarding how the actors were involved in developing the policy, the extent to which the phases in the process overlapped and the rigor of methods for searching and assessing evidence. There were also differences in core values, which influenced the choices regarding dissemination and implementation strategies (for more information about the similarities and differences, see Additional file 3).

## Discussion

**Fig. 2:**
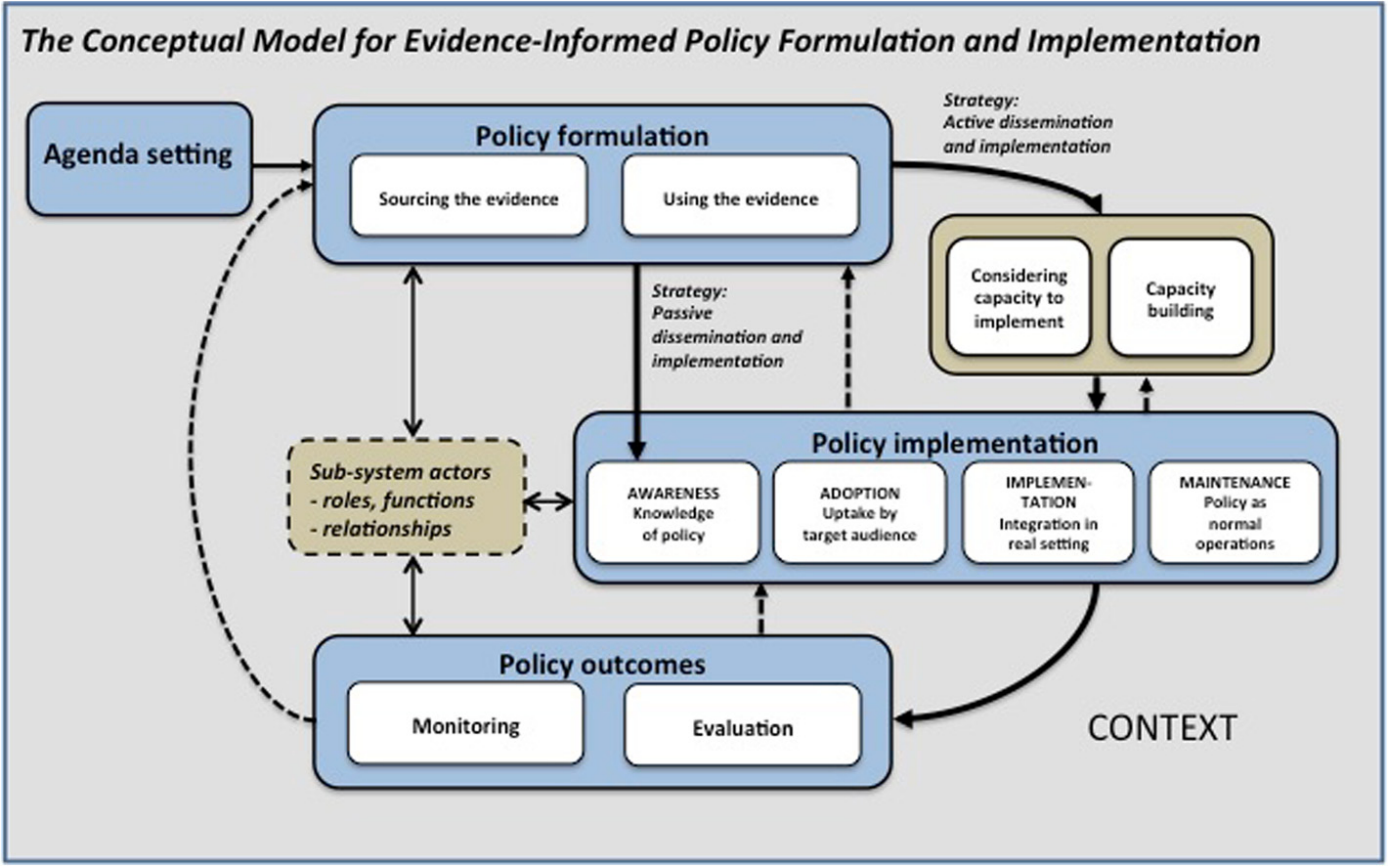
Revised Conceptual Model for Evidence-Informed Policy Formulation and Implementation

The two policies differed greatly regarding how evidence was used, how the policies were formulated, and the extent to which the policy phases overlapped. Similarities were an emphasis on capacity assessment and modification of activities based on the assessment and a highly active implementation approach relying on networks of stakeholders. We found that the Conceptual Model for Evidence-Informed Policy Formulation and Implementation (Fig. 1) was a useful tool for organizing the data. We also suggest some further development to the model based on new categories that emerged in the analysis (Fig. 2). These findings are discussed below, and implications for practice and research are suggested.

One of the main findings concerned how evidence was used in the formulation of the policies. What was regarded as evidence and how this knowledge was used depended heavily on the nature of the policy. The guidelines in Case 1 share the characteristics of a practice policy as presented by Black [23]. Practice policies are described as rather linear and rationalist processes for sourcing and using scientific evidence. The nature of the policy in Case 2 represents a combination of service and governance policies, implying a weaker relation between scientific evidence and the policy [23].

Other reasons behind differences in the use of evidence concerned policy actors’ beliefs and the coalitions that were formed (as described in the Advocacy Coalition Framework [15]). In Case 1, there was a central policy core belief shared by the principal actors that high-quality scientific evidence is the key to creating value for the patients. This case can be seen as an optimal model for developing evidence-based policy, in terms of using a systematic and transparent decision-making process using the best available research evidence [24, 25]. In Case 2, a central policy core belief was that knowledge that has been proven to work in practical settings is necessary in order to create value for the patients. A broad definition of evidence, including both scientific and practical knowledge such as expert opinions, values, and traditions [8] were used. The use of evidence in Case 2 is in line with prior studies suggesting that policymakers tend to interpret and use evidence in a broad sense [7]. The goal was to use a scientifically sound evidence base, even though this was done indirectly and in rather non-systematic ways. Thus, the policy in Case 2 was characterized by a pragmatic approach with regard to the policy content rather than strictly scientific as in Case 1. Case 2 is an example of a highly complex and political process of policy negotiation. These processes often consist of trade-offs between competing interests and values [26], which was also the case in this study. The two cases can be said to represent different positions, not only regarding the use of different sources of evidence but also in the contextualization of the policy. This has to do with the main actors’ values and credibility; NBHW cannot make policies if there is not enough high quality evidence, while SALAR cannot participate in policymaking that would violate the interests of its members, i.e., the regional and local authorities [27]. These results highlight the importance of analyzing coalitions’ sub-system actors and the roles, functions, and possible relationships between these actors.

The Advocacy Coalition Framework assumes that coalitions utilize resources to influence the policy process [16]. An important resource for the coalition in Case 1 was the supervisory authority of the government agency, even though it was not formally exercised in this case. Other resources were related to information and the involvement of researchers and clinical experts, which increased the scientific value of the guidelines. The support of key healthcare professional actors was mobilized through the health professional organizations. The main resources used in Case 2 were related to credibility and the ability to mobilize support from line managers and staff at local level trough higher-level managers. Moreover, the coalition had strong financial resources that were used for extensive implementation support and economic incentives. Resources related to formal authority were also used, since the politically governed interest organization had a formal agreement with the government about the policy.

Our findings also offer insight into the policymakers’ process of analyzing target audiences’ capacity to implement a policy and the actions taken based on this knowledge. In both cases, policymakers conducted analyses of capacity, concluded that the target groups lacked the capacity to act on the policy without support and thus initiated activities to strengthen the capacity. In Case 1, the government commissioned NBHW to organize implementation support, and in Case 2, substantial resources were allocated to national coordination of the implementation and to the establishment of support structures to facilitate regional and local implementation. We suggest the addition of a new element describing capacity-building to the Conceptual Model for Evidence-Informed Policy Formulation and Implementation.

In both cases, a highly active implementation approach was used, relying on collaboration between actors. In Case 1, NBHW took as its starting point that healthcare is highly professionalized and that influence and learning between health professionals would be effective for inducing change. In Case 2, SALAR’s aim was to strengthen the provider systems, structures for collaboration and work practices, thus seeking partnerships with regional and local managers. The partnership approach illustrates typical examples of soft law governance dealing with creating conditions for collective actions [14].

We found that the process in Case 1 followed the phases illustrated in the Conceptual Model for Evidence-Informed Policy Formulation and Implementation (Fig. 1). This implied a sequential process, starting with the development of the policy content, followed by the assessment of the capacity to implement and then the implementation activities. However, NBHW sought to address implementation determinants during policy formulation, for example by involving stakeholders. In Case 2, the policy formulation and implementation were integrated, implying that the content of the policy was developed iteratively while the policy was being implemented on a large scale. The policy outcomes were also continuously monitored and evaluated. One important feature of this policy process was the yearly renegotiation between the government and SALAR, where assessment of the past year’s policy outcomes and the implementation experiences provided input to the negotiation for the next agreement. This illustrates the emergent character of the policy and its implementation, a process that required flexibility and enabled corrective actions based on gathered knowledge. This process exemplifies both contextualizing of the policy content and governance. We suggest revisions to the Conceptual Model for Evidence-Informed Policy Formulation and Implementation that enables description and analysis of overlapping policy phases and iterative qualities of the process (dotted arrows in Fig. 2).

### Methodological considerations

A strength of the study is the use of two concurrent, empirical cases within the same country. Although the two cases are distinct, they help to highlight a spectrum of actors and resources involved in the formulation and implementation of evidence-informed health policy. The cases provided variation, which was important for drawing conclusions about the usefulness of the conceptual model. The longitudinal design and the use of multiple data collection methods increased the credibility of the findings. Another strength was to combine deductive and inductive approaches in the analysis by using a theoretical framework to organize the data, but still allowing new relevant categories to emerge. The national context limits the possibility to generalize to other nations, while the use of the conceptual model makes theoretical generalizations possible. Another potential limitation was the difference between the cases regarding the economic resources, which in some aspects presented a challenge to the comparison. In addition, we focused on sub-system actors at an institutional level rather than individual actors, and by doing so, we may not have captured influences by specific individuals. We were not able to study the policy outcomes and thus cannot draw any conclusions about the success of the policies.

## Conclusions

The policy actors’ roles and functions were found to have a great influence on the choices of strategies and collaborators in all policy phases. The Conceptual Model for Evidence-Informed Policy Formulation and Implementation was found to be a useful tool for organizing and analyzing the data. However, it provided insufficient guidance for analyzing actors involved in the policy process, capacity-building strategies, and overlapping policy phases. A revised version of the model that includes these aspects is suggested.

## Additional files

Additional file 1:
**Case descriptions.** Description of the problems, aims, content and the agencies involved in the two policy cases. (DOCX 133 kb) Additional file 2:
**Description of the study setting—Swedish health and social care.** Description of the setting for evidence-informed policymaking in Swedish health and social care. (DOCX 127 kb) Additional file 3:
**Key similarities and differences between the policy processes in the cases.** Table summarizing the key similarities and differences between the policy processes in the two cases. (DOCX 105 kb)

## Abbreviations

MHSA: Ministry of Health and Social Affairs
NBHW: National Board of Health and Welfare
SALAR: Swedish Association of Local Authorities and Regions
