# Supplementary material for: Evidence-informed policy formulation and implementation: a comparative case study of two national policies for improving health and social care in Sweden
Source: Implement Sci. 2015 Dec 8;10:169. doi: 10.1186/s13012-015-0359-1 (PMC4672562; doi:10.1186/s13012-015-0359-1)
Supplement: Additional file 1: — Case descriptions. Description of the problems, aims, content and the agencies involved in the two policy cases. (DOCX 133 kb) [file 13012_2015_359_MOESM1_ESM.docx]

Strehlenert, H., Richter-Sundberg, L., Nyström, M.E. and Hasson, H.: **Evidence-informed policy formulation and implementation: Comparative case study of two national policies for improving health and social care in Sweden**

# Additional file 1: Case descriptions

### Case 1: The national clinical guidelines for methods of preventing disease

*Problem.* Lifestyle habits like the use of tobacco, the hazardous use of alcohol, insufficient physical activity and unhealthy eating habits cause serious diseases such as cardiovascular disease, diabetes and cancer(Dodson, Elizabeth A., Brownson, Ross C., Weiss, Stephen, 2012). Disease prevention constitutes one of the Swedish healthcare system’s missions, defined in the Health and Medical Service Act, but efforts to prevent disease occupy only a minor place in the system.

*Aim.* The policy aimed to support healthcare leaders and professionals in prioritizing disease-preventive methods that are beneficial from a scientific, cost-effective and ethical perspective. The policy also aimed to guide healthcare decision-makers in resource allocations for health professionals towards interventions targeted to change life-style habits associated with increased risk of disease.

*Main agencies involved.* The government, represented by the Ministry of Health and Social Affairs, commissioned the autonomous government agency the National Board of Health and Welfare (NBHW) to develop the guidelines. NBHW has the mandate to support and exercise public authority in fields such as social, health and medical services, and one of its key functions is to collect, compile, analyze and disseminate evidence-based information to care providers. The development of national clinical guidelines is a central process in this assignment.

*Content*. National clinical guidelines are recommendations to optimize patient care based on a systematic review of evidence and an assessment of the benefits and harms of alternative care options [1]. National clinical guidelines are not legally binding but rather explicit and voluntary rules [2]. The guidelines included recommendations concerning disease preventive interventions and definitions of key concepts to enable identification of policy target populations and evaluation. The policy provided scientific evidence supporting the interventions and an explicit description of how the policy was developed. For more in depth information on the development and implementation of the guidelines see [3, 4].

### Case 2: Agreement on coordinated health and social care for the most ill older people

*Problem.* The proportion of older people is increasing. Longer life expectancy implies that there will be more people suffering from complex health problems in old age and thus that the need for health and social care will increase. Older people do not receive adequate care, due to a lack of evidence-based practices and poor coordination of care [5].

*Aim.* The policy aimed to improve the quality and coordination of care for older people with complex health problems and to streamline the use of resources, so that the care will have a stronger focus on preventive measures and be based to a greater extent on evidence and patient needs.

*Main agencies involved.* The government, represented by the Ministry of Health and Social Affairs, negotiated the policy with the Swedish Association of Local Authorities and Regions (SALAR). SALAR supports its members through advice, education and coordination, and represents the regional and local authorities in negotiations with the government. In practice, it has been given a strong position to negotiate healthcare policies and to act as a “veto player” in policymaking [6]. However, SALAR has no direct authority and cannot sanction its members. NBHW was also involved in the overall work within the older people area, and was responsible for assessing regional and local authorities’ performance on policy related indicators.

*Content*. The policy was based on a framework agreement between the government and SALAR and comprised five improvement areas: preventive care, palliative care, dementia care, pharmacological treatment and coordination of care. The regional and local authorities that achieved the set objectives were awarded with performance-based grants. Funds were allocated for improvement coaches, management support, development of quality registries and analysis work to support regional and local authorities in developing joint, systematic processes for improvement work. A more thorough presentation about the policy has been made previously [7].

## References

1. Institute of Medicine: *Clinical Practice Guidelines We Can Trust*. National Academies Press; 2011.

2. Mörth U: *Soft Law in Governance and Regulation: An Interdisciplinary Analysis*. Cheltenham: Edward Elgar Publishing; 2004.

3. Richter-Sundberg L, Kardakis T, Weinehall L, Garvare R, Nyström ME: **Addressing implementation challenges during guideline development: A case study of Swedish national guidelines for methods of preventing disease**. *BMC Health Serv Res* 2015, **15**:19.

4. Kardakis T, Weinehall L, Jerdén L, Nyström ME, Johansson H: **Lifestyle interventions in primary health care: professional and organizational challenges.** *Eur J Public Health* 2014, **24**:79–84.

5. Gurner U, Thorslund M: *Dirigent Saknas I Vård Och Omsorg För Äldre: Om Nödvändigheten Av Samordning*. Stockholm: Natur och Kultur; 2003.

6. Fredriksson M: **Between Equity and Local Autonomy: A Governance Dilemma in Swedish Healthcare**. Uppsala University; 2012.

7. Nyström ME, Strehlenert H, Hansson J, Hasson H: **Strategies to facilitate implementation and sustainability of large system transformations: a case study of a national program for improving quality of care for elderly people.** *BMC Health Serv Res* 2014, **14**:401.
