# Supplementary material for: Evidence-informed policy formulation and implementation: a comparative case study of two national policies for improving health and social care in Sweden
Source: Implement Sci. 2015 Dec 8;10:169. doi: 10.1186/s13012-015-0359-1 (PMC4672562; doi:10.1186/s13012-015-0359-1)
Supplement: Additional file 2: — Description of the study setting—Swedish health and social care. Description of the setting for evidence-informed policymaking in Swedish health and social care. (DOCX 127 kb) [file 13012_2015_359_MOESM2_ESM.docx]

Strehlenert, H., Richter-Sundberg, L., Nyström, M.E. and Hasson, H.: **Evidence-informed policy formulation and implementation: Comparative case study of two national policies for improving health and social care in Sweden**

# Additional file 2: Description of the study setting - Swedish health and social care

The Swedish healthcare system is mainly tax-funded. Health and social care policy is the responsibility of the central government, while the provision of care is decentralized to autonomous regional and local authorities with their own democratic decision bodies. There are 21 regional authorities responsible for delivering primary care and specialized healthcare, and 290 local authorities providing social care, older people care and home healthcare. This decentralization implies strong positions for these authorities in policymaking [1]. Over the past decades, the idea of evidence-based policymaking has emerged in the light of New Public Management. Initially, the general aim was to improve the efficiency based on systematic knowledge, but evidence-based policymaking is now seen as form of governance where the government explicitly aims to influence healthcare professionals. National clinical guidelines are examples of evidence-based policies that aim to guide the practice [2, 3]. The current development is towards stimulating performance and results rather than activities and projects. The government nowadays negotiates policies with the regional and local authorities’ interest organization (the Swedish Association of Local Authorities and Regions, SALAR) to improve specific areas within health and social care. In addition, targeted government grants with performance-based bonuses were introduced in 2006. The use of this combination of instruments has increased over the past decade. The main drivers in this development were the government’s ambitions to monitor and manage the effects of government grants and to create a common agenda for development between the government, regional and local authorities [4].

## References

1. Vrangbaeck K: **The political process of restructuring Nordic health systems**. In *Nordic Health Care Systems: Recent Reforms And Current Policy Challenges: Recent Reforms and Current Policy Challenges*. Edited by Magnussen J, Vrangbaeck K, Saltman RB. Maidenhead: Open University Press; 2009.

2. Fredriksson M, Blomqvist P, Winblad U: **Recentralizing healthcare through evidence-based guidelines – striving for national equity in Sweden**. *BMC Health Serv Res* 2014, **14**:509.

3. Alm M: **When knowledge is the ruling force – on organizational and professional conditions for knowledge governance in substance abuse treatment**. Linnaeus University; 2015.

4. The Swedish Agency for Health and Care Services Analysis: *Statens Styrning Av Vården Och Omsorgen Med Prestationsbaserad Ersättning*. Stockholm; 2013.
