# Supplementary material for: Evidence-informed policy formulation and implementation: a comparative case study of two national policies for improving health and social care in Sweden
Source: Implement Sci. 2015 Dec 8;10:169. doi: 10.1186/s13012-015-0359-1 (PMC4672562; doi:10.1186/s13012-015-0359-1)
Supplement: Additional file 3: — Key similarities and differences between the policy processes in the cases. Table summarizing the key similarities and differences between the policy processes in the two cases. (DOCX 105 kb) [file 13012_2015_359_MOESM3_ESM.docx]

Strehlenert, H., Richter-Sundberg, L., Nyström, M.E. and Hasson, H.: **Evidence-informed policy formulation and implementation: Comparative case study of two national policies for improving health and social care in Sweden**

Additional file 3: Key similarities and differences between the policy processes in the cases

Case 1: The national clinical guidelines for methods of preventing disease

Case 2: Agreement on coordinated health and social care for the most ill older people

|  | **Similarities** | **Differences** |
| --- | --- | --- |
| **Policy formulation** |  | *Case 1:* Policy was developed independently by the government agency (NBHW).  *Case 2:* Policy was developed in negotiations between the government and SALAR.  *Case 1:* Policy was finalized prior to implementation.  *Case 2:* Policy was developed iteratively and was re-negotiated each year, i.e. while being implemented.  *Case 1:* Pre-defined, systematic methods were used for searching and assessing scientific evidence.  *Case 2:* Pragmatic and exploratory approach was used in searching and assessing scientific and non-scientific evidence. |
| **Strategy for dissemination and implementation** |  | *Case 1:* Evidence and professional knowledge were seen as central.  *Case 2:* Value for the patient and organizational factors were seen as central.  *Case 1:* Health professional organizations were used for dissemination and implementation.  *Case 2:* Managers, designated regional support structures, (e.g. improvement coaches) and the national quality registries were engaged in dissemination and implementation. |
| **Considering capacity to implement** | Assessments were made of target audiences’ implementation capacity.  Results influenced decisions about implementation support. |  |
| **Policy implementation and maintenance** | Existing channels were used for dissemination.  Arenas were created for sharing experiences and supporting regional and local implementers.  Interactive educational activities were arranged. | *Case 1:* NBHW provided funding to independent implementation projects conducted by health professional organizations. No performance-based grants.  *Case 2:* SALAR led and coordinated the national implementation. Performance-based grants were used as incentives during the implementation.  *Case 1:* Policy will be updated and disseminated every three to five years.  *Case 2:* Policy will not be updated after the implementation. |
| **Policy outcomes** | Indicators were identified for monitoring, feedback and comparison of results during and after the implementation. |  |

## List of abbreviations

NBHW - National Board of Health and Welfare

SALAR - Swedish Association of Local Authorities and Regions
